# Optical Coherence Tomography Biomarkers in Predicting Treatment Outcomes of Diabetic Macular Edema After Dexamethasone Implants

**DOI:** 10.3389/fmed.2022.852022

**Published:** 2022-06-09

**Authors:** Yu-Te Huang, Yen-Chieh Chang, Ping-Ping Meng, Chun-Ju Lin, Chun-Ting Lai, Ning-Yi Hsia, Huan-Sheng Chen, Peng-Tai Tien, Henry Bair, Jane-Ming Lin, Wen-Lu Chen, Yi-Yu Tsai

**Affiliations:** ^1^Department of Ophthalmology, China Medical University Hospital, China Medical University, Taichung, Taiwan; ^2^School of Medicine, College of Medicine, China Medical University, Taichung, Taiwan; ^3^Department of Optometry, Asia University, Taichung, Taiwan; ^4^An-Shin Dialysis Center, NephroCare Ltd., Fresenius Medical Care, Taichung, Taiwan; ^5^Graduate Institute of Clinical Medical Science, College of Medicine, China Medical University, Taichung, Taiwan; ^6^Byers Eye Institute, Stanford University School of Medicine, Stanford, CA, United States

**Keywords:** diabetic macular edema (DME), disorganization of retinal inner layers, hyperreflective foci, intravitreal dexamethasone implant, optical coherence tomography biomarkers, subretinal fluid

## Abstract

**Purpose:**

To identify optical coherence tomography (OCT) biomarkers that may predict functional and anatomical outcomes in diabetic macular edema (DME) patients treated with intravitreal dexamethasone (DEX) implant.

**Materials and Methods:**

Sixty-four eyes from 50 patients with DME were enrolled. Best-corrected visual acuity (BCVA) and OCT biomarkers including central retinal thickness (CRT), subretinal fluid (SRF), intraretinal cysts (IRC), ellipsoid zone disruption (EZD), disorganization of retinal inner layers (DRIL), hard exudate (HE), hyperreflective foci (HRF), epiretinal membrane (ERM), and vitreomacular interface (VMI) changes were evaluated at baseline and at 3, 6, and 12 months after treatment. Multiple logistic analysis was performed to evaluate each OCT biomarker as a predictive factor for functional and anatomical improvement at the end of treatment.

**Results:**

The presence of SRF at baseline was associated with a favorable outcome, with CRT improving by more than 100 μm after treatment from multivariate logistic regression analysis [odds ratio 6.16 (1.75–21.6)]. In addition, baseline SRF predicted a greater CRT improvement from multiple regression analysis (model R-square 0.11, *p* = 0.006). The reduction of DRIL, SRF, LONLC, IRC, and EZD were correlated with better CRT improvement (more than 100 μm) (*P* < 0.05). SRF and EZD recovery can also predict better visual prognosis (*P* < 0.05).

**Conclusion:**

OCT biomarkers can be used to predict who may benefit the most after DEX treatment. We suggest that the DEX implant should be considered as a first line treatment in DME patients with SRF.

## Introduction

Diabetes mellitus (DM) is one of the most important global health issues, with an estimated 425 million patients suffering from DM worldwide (1). Among the complications of DM, diabetic macular edema (DME) is the most frequent cause of visual impairment, with prevalence rates ranging from 7 to 12.8% among those with diabetes from different population-based studies (2–4). DME is also one of the leading complications among those with retinal vascular disorders (5).

Antivascular endothelial growth factor (anti-VEGF) injections are generally considered over focal grid laser photocoagulation as the gold standard and first-line therapy for clinically significant DME (6, 7). Nonetheless, up to 40% of patients do not respond optimally, with half of that classified as non-responder after monthly injections for 1 year (8).

Besides anti-VEGFs, there are other available treatments for DME including laser, surgery, and corticosteroids, with each targeting different pathogenic mechanisms of the disease. The biodegradable intravitreal dexamethasone (DEX) implant (Ozurdex^®^, Allergan, Inc., Irvine, CA, United States) has been identified as an effective treatment of DME and is approved by the US Food and Drug Administration (FDA) (9). In addition, the beneficial effects of DEX implant in anti-VEGF non-responders are well-established in several studies (9, 10).

As it is crucial to identify which DME patients may most benefit from DEX implant, spectral domain optical coherence tomography (SD-OCT) may serve as non-invasive, rapid, safe, and cost-effective predictive tool. Quantitative measurements in OCT such as central retinal thickness (CRT) and qualitative data i.e., different space of fluid distribution, disorganization of the retinal inner layers (DRIL), ellipsoid zone disruption (EZD), hard exudate (HE), hyperreflective foci (HRF) may serve as OCT biomarkers (11–13). The aim of this study is to investigate whether these OCT biomarkers can serve as predictors to identify which DME patients will most benefit from DEX implants.

## Materials and Methods

This retrospective, interventional case series study was conducted at China Medical University Hospital (CMUH) between January 2018 and January 2021. The study was performed in accordance with the World Medical Association’s Declaration of Helsinki and the study design was approved by the Institutional Review Board of CMUH (IRB number: CMUH109-REC3-158). Owing to the retrospective design of the study, the review board waived the need for written informed consent.

### Population and Study Design

Inclusion criteria were as follows: (1) age older than 18 years; (2) history of type 1 or 2 diabetes mellitus; (3) presence of severe NPDR (non-proliferative diabetic retinopathy) and PDR (proliferative diabetic retinopathy) confirmed by widefield fluorescein angiography; (4) DME with central retinal thickness more than 250 mm; (5) treatment with at least one DEX implant; (6) follow-up lasting at least 3 months. For patients who received bilateral treatment with DEX, both eyes could be included.

The main exclusion criteria were as follows: (1) a history of pars plana vitrectomy in the study eye; (2) concomitant glaucoma; (3) another concomitant retinal disease that causes macular edema including retinal vein occlusion, neovascular age-related macular degeneration or uveitis; (4) previous treatment with intraocular anti-VEGF within 3 months or corticosteroids within 6 months prior to treatment with DEX implant; (5) any other ocular condition that can influence visual acuity.

Each patient’s demographic data, medical history (including diabetes and hypertension), best-corrected visual acuity (BCVA), IOP, and CRT, as well as the occurrence of any complications, prior to and 3, 6, and 12 months after the DEX implant were retrieved from the electronic medical record. Any patients whose IOP exceeded 25 mmHg at any visit was evaluated and treated accordingly. Patients were eligible for retreatment with DEX implant if their retinal thickness increased by 50 μm from the lowest recorded level, and further doses of DEX implant were also given if the patient experienced a recurrence of ME as determined by OCT.

### Outcome Measurement

**FIGURE 1:**
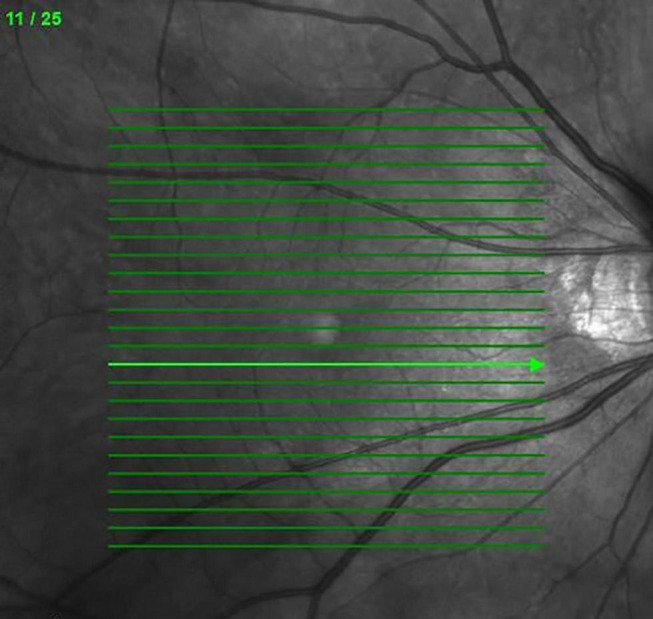
The 25 6-mm radial scans across the retina centered on the fovea in SD-OCT.

The OCT scans were all obtained by SD-OCT (Heidelberg Spectralis, Heidelberg, Germany). In each patient, SD-OCT was used to record 25 6-mm radial scans across the retina centered on the fovea (6 × 6 mm area) (Figure 1). Grading of OCT images was all performed by two evaluators manually (YTH and PPM), masked to details of clinical findings and systemic parameters. When disagreement occurred, a third senior retina specialist would determine the final grading (CJL). OCT images were graded for the following parameters at each visit (baseline, 3, 6, and 12 months): EZD, DRIL, presence of HE, presence of HRF, its quantity (average number in one cut), and location (between the ILM and the INL; between the OPL and the ELM; in all retinal layers), SRF, intraretinal cyst (IRC), presence of an epiretinal membrane (ERM), presence of large outer nuclear layer cyst (more than 100 μm) (LONLC) and vitreomacular interface (VMI) [posterior vitreous detachment (PVD), vitreomacular adhesion (VMA), and vitreomacular traction (VMT)].

### Statistical Analysis

All analyses were computed by using PASW Statistics 18 software (Version 18.0. Chicago: SPSS Inc.). The numerical data are expressed as mean and standard deviation and the categorical variables as absolute frequency and percentage. The baseline characteristic of patients and changes in CRT were analyzed by using Chi-square and one-way ANOVA. Multiple regression analysis was performed to evaluate the possible OCT biomarker (EZD, DRIL, HE, HRF, SRF, IRC, ERM, and VMI) as predictive factors for final visual acuity improvement at the end of treatment. A *p*-value smaller than 0.05 was considered to be statistically significant.

## Results

### Study Population

A total of 64 eyes from 50 participants were ultimately included in this study. Demographic and baseline characteristics are detailed in Table 1. Of the included eyes with DME, 37 (58.8%) were naïve and 27 (42.2%) were refractory to previous anti-VEGF injections; 59 (92.2%) of patients had severe NPDR and 5 (7.81%) patients had PDR. Thirty patients (60%) were female, 20 (40%) were male, and mean age was 66.22 ± 10.17 years old. Mean duration of follow-up was 9.89 ± 3.24 months and 92.2% of the cases were followed up more than 6 months. A total of 38 eyes (59.4%) were phakic and 26 eyes (40.6%) were pseudophakic. HbA1c levels were available for 29 patients; the mean value was 7.47 ± 1.34%.

**TABLE 1:** Baseline clinical data and status of OCT biomarkers.

Baseline clinical data	50 patients, 64 eyes
Age	66.22 ± 10.17
Gender (female)	30/50 (60.0%)
Lens (pseudophakic)	26/64 (40.6%)
Side (OD)	30/64 (46.9%)
HbA1c	7.47 ± 1.34
CRT_*initial*_	411.17 ± 119.50
LogMAR_*initial*_	0.81 ± 0.46
IOP_*initial*_	15.66 ± 3.40
s/p IVI	27/64 (42.2%)
s/p PST	28/64 (43.8%)
s/p PRP	37/64 (57.8%)
**Follow-up months**	
3	5/64 (7.8%)
6	15/64 (23.4%)
12	44/64 (68.8%)
Mean	9.89 ± 3.24
**Injection times**	
1	21/64 (32.8%)
2	23/64 (35.9%)
3	20/64 (31.3%)
Mean	1.98 ± 0.81

### Anatomical and Functional Outcome

**FIGURE 2:**
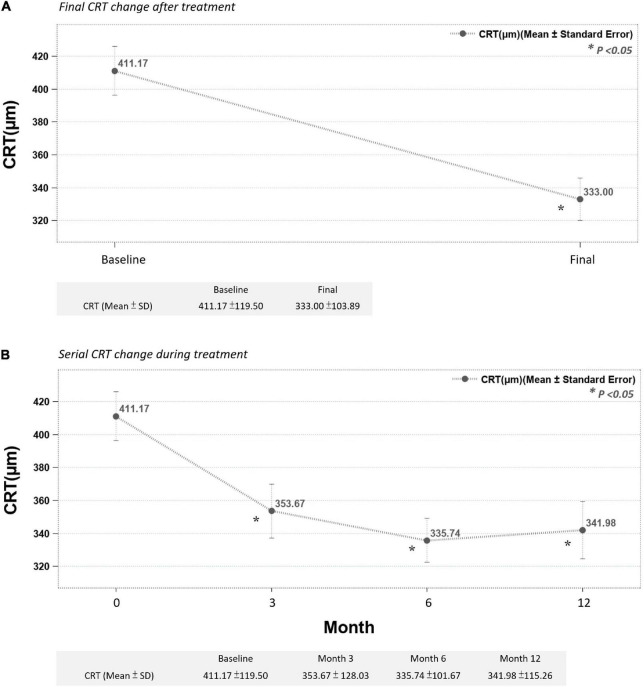
**(A)** Final CRT significantly improved after treatment. **(B)** Mean CRT improved significantly after the third month and continuously improved up to the end of the study (month 12) (*p < 0.05 compared to before-treatment data).

**FIGURE 3:**
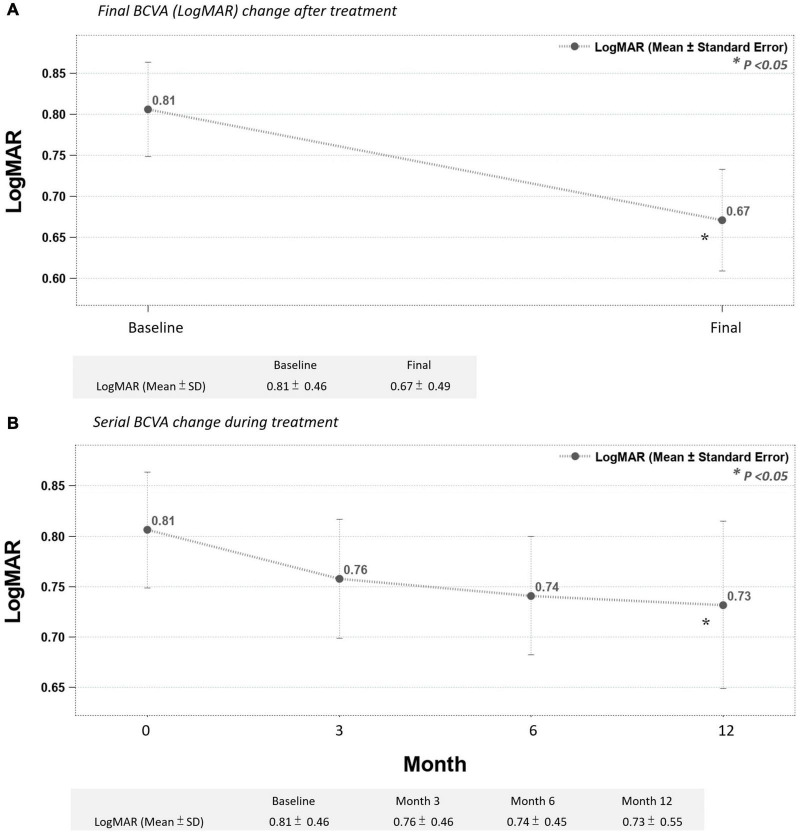
**(A)** Final BCVA (LogMAR) significantly improved after treatment. **(B)** Mean BCVA improved gradually after the third month, reaching statistical significance in month 12 (*p < 0.05 compared to before-treatment data).

The mean final change in CRT for all 64 eyes after the DEX implant treatments reached a statistically significant level (decreased from a mean initial CRT of 411.17 ± 119.50 μm to a mean final CRT of 333.00 ± 103.89 μm, *p* < 0.05) (Figure 2A). During the follow-up period, CRT showed rapid improvement in the first 3 months, then fluctuated within a stable range (Figure 2B). The mean change in LogMAR BCVA of all 64 eyes after the DEX implant treatments showed statistical significance (0.81 ± 0.46–0.67 ± 0.49, *p* < 0.05). The BCVA improved gradually but significantly after 12 months (Figure 3), taking slightly longer to reach the level of significance than the CRT changes.

**FIGURE 4:**
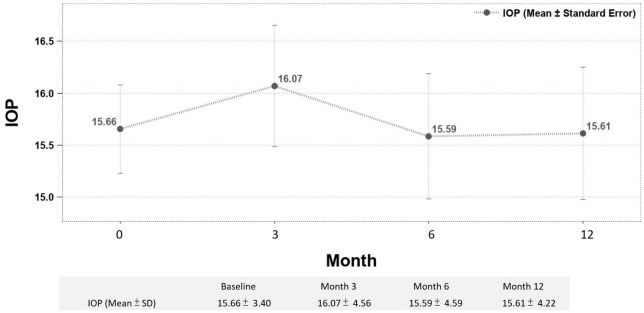
Serial IOP change during treatment, revealing no obvious elevation.

During the follow-up period, 21 (32.8%) eyes received one injection. 23 (35.9%) eyes received two injections, and 20 (31.3%) eyes received three injections (mean injection number: 1.98 ± 0.81). IOP elevation is an important concern in patients receiving DEX implant. The mean change IOP of all the eyes were from 15.66 ± 3.40 to 15.89 ± 4.83 mmHg, which showed no statistically significant difference (*p* > 0.05). Serial IOP measurements during treatment also revealed no obvious elevation (Figure 4).

### Optical Coherence Tomography Biomarker Analysis

In our study group, baseline OCT biomarker characteristics showed high prevalence of HRF (92.2%), IRC (82.8%), DRIL (79.7%), and HE (71.9%); and lower prevalence of SRF (23.4%) and LONLC (31.3%) (Table 2).

**TABLE 2:** Baseline OCT Biomarkers.

**Baseline OCT biomarkers**	
DRIL (+)	51/64 (79.7%)
ERM (+)	35/64 (54.7%)
EZD (+)	34/64 (53.1%)
HE (+)	46/64 (71.9%)
HRF (+)	59/64 (92.2%)
IRC (+)	53/64 (82.8%)
LONLC (+)	20/64 (31.3%)
SRF (+)	15/64 (23.4%)
VMI (VMA or VMT)	16/64 (25.0%)

**FIGURE 5:**
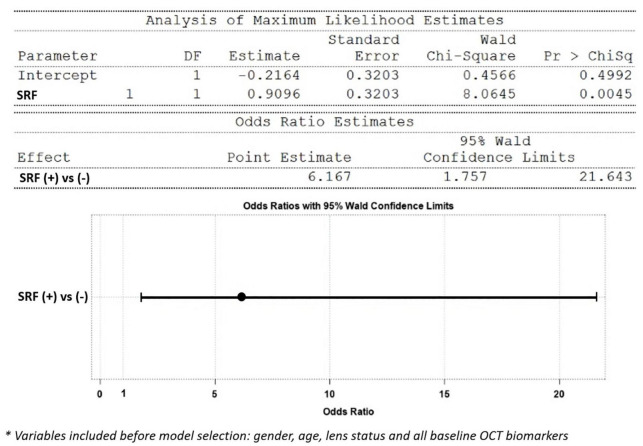
Model using OCT biomarkers as predictors of CRT improvement greater than 100 μm after treatment (multivariate logistic regression): the presence of SRF at baseline favored an outcome with CRT improvement > 100 μm [SRF (+) vs. (-): odds ratio 6.16 (1.75–21.6)].

Multiple regression analysis was used for evaluating possible OCT biomarkers that predict improvement of CRT at the end of treatment. The presence of SRF at baseline was correlated with CRT improvement of more than 100μm after treatment from multivariate logistic regression analysis [with SRF vs. without SRF: odds ratio 6.16 (1.75–21.6)] (Figure 5). Also, in Table 3, positive baseline SRF predicted a greater CRT improvement from multiple regression analysis (model R-square 0.11, *p* = 0.006).

**TABLE 3:** Model using OCT biomarkers as predictors to predict extent of CRT improvement (ΔCRT) after treatment (multiple regression).

Analysis of variance						

Source	DF	Sum of squares	Mean square	AIC	*F*-value	Pr > F
Model	1	122,153	122,153	–151.4508	8.01	0.0063
Error	62	945,646	15,252	–165.4669		
Corrected Total	63	1,067,799	0.4375	–201.8591		

**Variable**		**Parameter estimate**	**Standard error**	**Type II SS**	***F*-value**	**Pr > F**

Intercept		–54.00000	17.64292	142,884	9.37	0.0033
SRF		–103.13333	36.44305	122,153	8.01	0.0063

*Variables included before model selection: gender, age, lens status, and all baseline OCT biomarkers.

No single OCT biomarker at baseline was associated with BCVA improvement to a statistically significant degree (all *p* > 0.05). However, trends of BCVA improvements were more prominent in eyes without DRIL (69.2% eye without DRIL displayed BCVA improvement vs. 47.1% of eyes with DRIL.), and with HRF (54.2 vs. 20%) and PVD (56.3 vs. 37.5%) (Table 4).

**TABLE 4:** Treatment results of BCVA grouped by baseline OCT biomarker status.

	Percentage of eyes with BCVA improvement		Final BCVA response (Δ LogMAR, mean ± SD)	
Baseline biomarker	(+)	(–)	(+)	(–)
DRIL	24/51 (47.1%)	9/13 (69.2%)	–0.11 ± 0.43	–0.22 ± 0.21
ERM	19/35 (54.3%)	14/29 (48.3%)	–0.12 ± 0.43	–0.15 ± 0.35
EZD	17/34 (50.0%)	16/30 (53.3%)	–0.20 ± 0.44	–0.06 ± 0.33
HE	24/46 (52.2%)	9/18 (50.0%)	–0.14 ± 0.43	–0.11 ± 0.28
HRF	32/59 (54.2%)	1/5 (20.0%)	–0.15 ± 0.41	–0.00 ± 0.06
IRC	28/53 (52.8%)	5/11 (45.5%)	–0.14 ± 0.43	–0.09 ± 0.16
LONLC	9/20 (45.0%)	24/44 (54.5%)	–0.14 ± 0.52	–0.13 ± 0.33
SRF	6/15 (40.0%)	27/49 (55.1%)	–0.16 ± 0.44	–0.13 ± 0.38
VMI	6/16 (37.5%)	27/48 (56.3%)	–0.09 ± 0.33	–0.15 ± 0.41

*Comparing positive baseline biomarker status to negative baseline biomarker status, by Chi-Square and Student t-test.*

The DEX implant treatment significantly decreased the proportion of DRIL, SRF, LONLC, IRC and EZD (*p* < 0.05). In subgroup analyses, we investigated OCT biomarker changes correlated with final CRT improvements (greater than 100 μm or less than 100 μm) and final BCVA (VA improved group vs. VA not improved group). In the group with final CRT improvements greater than 100 μm, DRIL, SRF, LONLC, IRC, and EZD all significantly decreased while none of these OCT biomarkers significantly changed in groups with CRT improvements less than 100 μm (Table 5). In groups stratified by BCVA improvements, DRIL decreased regardless of VA status, while BCVA improvements were correlated with SRF and EZD resolution (*p* < 0.05) (Table 6).

**TABLE 5:** Changes of OCT biomarkers presentation pre (baseline) and after study in whole group and by final CRT response.

	Whole group		Group by final CRT response			
			CRT improved > 100 μm		CRT improved ≤ 100 μm	
	*Pre*	*After*	*Pre*	*After*	*Pre*	*After*
DRIL	51/64 (79.7%)	33/64 (51.6%)*	20/22 (90.9%)	10/22 (45.5%)*	31/42 (73.8%)	23/42 (54.8%)
SRF	15/64 (23.4%)	5/64 (7.8%)*	10/22 (45.5%)	0/22 (0.0%)*	5/42 (11.9%)	5/42 (11.9%)
LONLC	20/64 (31.3%)	7/64 (10.9%)*	10/22 (45.5%)	0/22 (0.0%)*	10/42 (23.8%)	7/42 (16.7%)
IRC	53/64 (82.8%)	42/64 (65.6%)*	21/22 (95.5%)	16/22 (72.7%)*	32/42 (76.2%)	26/42 (61.9%)
EZD	34/64 (53.1%)	25/64 (39.1%)	17/22 (77.3%)	10/22 (45.5%)*	17/42 (40.5%)	15/42 (35.7%)

*p < 0.05, compared to pre-study, by Chi-Square test.

**TABLE 6:** Changes of OCT biomarkers presentation in groups and by final BCVA response.

	Group by final BCVA response			
	VA improved (Δ LogMAR < 0)		VA not improved (Δ LogMAR ≥ 0)	
	*Pre*	*After*	*Pre*	*After*
DRIL	22/33 (72.7%)	15/33 (45.5%)*	27/31 (87.1%)	18/31 (58.1%)*
SRF	6/33 (18.2%)	1/33 (3.0%)*	9/31 (29.0%)	4/31 (12.9%)
LONLC	9/33 (27.3%)	3/33 (9.1%)	11/31 (35.5%)	4/31 (12.9%)*
IRC	28/33 (84.9%)	22/33 (66.7%)	25/31 (80.7%)	20/31 (64.5%)
EZD	17/33 (51.5%)	9/33 (27.3%)*	17/31 (54.8%)	16/31 (51.6%)

*p < 0.05, compared to pre-study, by Chi-Square test.

## Discussion

In the present study, we found the presence of SRF as the best OCT biomarker to predict CRT improvement. Also, the reduction of DRIL, SRF, LONLC, IRC, and EZD were correlated with better CRT improvement (more than 100 μm) (*P* < 0.05). SRF and EZD recovery can also predict better visual improvement.

Also, we demonstrated that under real-world conditions, DEX implant served as an effective and efficient strategy in treating DME, both anatomically and functionally. With an average of fewer than two injections, the treatment effect was sustained during the 1-year follow-up in most of the cases.

In practice, most clinicians consider anti-VEGF as a first-line treatment with laser photocoagulation as adjuvant therapy (14). DEX implant is mainly used as a second line therapy in most clinical situations due to the side effects of glaucoma and cataract progression (9, 15). Several guidelines recommend of DEX implant usage in patients with history of major cardiovascular event, vitrectomized eye, anti-VEGF non-responder, and pseudophakia (15–17). In the current study, we proposed qualitative OCT biomarkers in DME could provide important information to guide the treatment choice.

To date, there is limited literature focusing on the relationship between DEX implant efficacy, baseline OCT characteristics, and changes of OCT biomarkers in DME patients. Most studies focus on baseline OCT biomarkers as predictors of functional outcome. Zur et al. demonstrated that baseline continuous IS-OS layer and submacular fluid responded better to DEX implants. Also, treatment response was not different among treatment-native and refractory group (13). Meduri et al. proposed similar conclusions that the presence of SRF and the integrity of EZ were positive biomarkers in predicting the efficacy of DEX implant in treatment-naive DME patients (18). Vujosevic et al. showed that treatment-naive DME patients with baseline SRF had a better response to intravitreal dexamethasone rather than to anti-VEGF (19).

The presence of SRF in DME is associated with active inflammation and higher levels of IL-6 in vitreous cavity (20). Corticosteroids are well-known for its efficient anti-inflammation effects, with strong inhibition of TNF-α, VEGF, and. ICAM-1, and upregulation of anti-inflammatory agents such as adenosine and IL-10 (21). Therefore, as expected, the presence of baseline SRF severed as a good indicator for response to DEX implant treatment.

In contrast, the role of baseline HRF is less clear, with some studies considering it as a biomarker for improved DEX implant treatment response (19, 22) and other studies coming to the opposite conclusion, considering the lack of HRF as a better prognostic factor (15, 23). In our current study, the baseline HRF is a neutral OCT biomarker, with no statistically significant prediction of functional and anatomical outcomes, which is similar to the study by Ahn et al. (24). Pathology studies have suggested HRF to be lipoprotein extravasation after breakdown of the inner blood retinal barrier in the initial stages of the development of intraretinal hard exudates (25). Further studies are needed to identify its role in DME. In our study, higher DR severity may be another reason that baseline HRF had less predictive power for functional and anatomical outcomes. Also, the high percentage of HRF at baseline can be another evidence of high severity (92.2%). In fact, 56.25% (36/64) of the cases showed decreasing amounts of HRF.

In the present study, the reduction of DRIL, LONLC, IRC, and EZD was correlated with more than 100 μm CRT improvement. The presence of DRIL in DME was thought of as a sign of chronicity of macular edema and dysfunction of Muller cells (11). We hypothesized that reduction of DRIL after DEX implant might be related to its anti-inflammatory effect on Muller cells, correlating with more CRT improvement. The reduction of other parameters like LONLC and IRC was more apparently related to better anatomical outcome with less space occupied fluid inside the retina. In addition, EZD indicated the breakdown of photoreceptors cells, hindered the normal visual phototransduction and was related to the worse functional outcome (26). We did not find other parameters like ERM, VMI, and the quality and quantity of HRF to be related the visual and anatomical prognosis in the present study.

Central macular thickness is a well-established proxy for treatment outcome in several studies (14–19). However, in our study, there are some gaps between anatomical and functional outcomes. There are some possible explanations. First, we only enrolled DME patients who were confirmed by OCT, with severity indicated by the high presence of IRC, HRF, and DRIL (all near 80%). Also, the use of a DEX implant was considered as second choice in most clinical situations, with 57.8, 43.8, and 42.2% cases having been previously treated with panretinal photocoagulation (PRP), posterior subtenon triamcinolone injection (PST), and anti-VEGF respectively. Combined with the chronicity and high severity of our DME patients, limited visual recovery may occur despite an improved foveal contour. As a results, the findings of our study are still valid if DEX implants are used in an earlier stage or in treatment-naïve patients.

The progression of cataract might bias visual acuity improvement despite successful treatment with DEX implant. Indeed, there would be some cataract progression in patients who received more than one DEX implant. But we are convinced the outcomes are valid for several reasons. First, there was no significant cataract progression that required operation during the follow-up period. More importantly, we performed multivariate analysis and lens status was not significantly associated with outcomes. Previous studies have found similar conclusions that the lens status was not significantly associated with differences in BCVA (10, 27). Lastly, if we had divided our subjects into two groups, the smaller case number in each group would make the outcomes less reliable.

About sample size, because this is a retrospective study, we have collected all the available and eligible cases as many as possible. In the meantime, we did not set up a minimally required sample size because the main purpose of this study is not to compare outcomes between exposure and non-exposure groups for which sample size is crucial in the study design to make sure the power of the study is enough to confirm the value of intervention. Instead, the nature of our study is an exploratory one, which focused on the presentation of OCT biomarkers in patients with different characteristics, their changes during the treatment process, their role of prediction to treatment response and so on.

Most of the limitations of this study came from its retrospective nature. And the clinical decision to initiate DEX implant was based on the physician’s choice. Moreover, due to the strict inclusion and exclusion criteria, there were a relatively low number of study subjects. Also, we could not separately treat naïve patient from the non-naïve patients. However, we provided the wash-out period (intraocular anti-VEGF within 3 months or corticosteroids within 6 months). Wide field fluorescein angiographies were not analyzed, therefore information regarding macular perfusion and peripheral non-ischemia area was not available.

In conclusion, OCT biomarkers can be used to guide selection of DME patients who may most benefit from DEX implants. We furthermore suggest that DEX implant should be considered as a first-line treatment in patient with SRF at baseline. Changes of DRIL, SRF, LONLC, IRC, and EZD can also help predict the treatment response in DEX implant. Further head-to-head large-scale clinical trial between anti-VEGF agents and DEX implant is needed to identify the role of these OCT biomarkers to optimized current treatment of DME patients.

## Data Availability Statement

The raw data supporting the conclusions of this article will be made available by the authors, without undue reservation.

## Ethics Statement

The studies involving human participants were reviewed and approved by the China Medical University Hospital. The ethics committee waived the requirement of written informed consent for participation.

## Author Contributions

All authors listed have made a substantial, direct, and intellectual contribution to the work, and approved it for publication.

## Conflict of Interest

H-SC was employed by NephroCare Ltd. The remaining authors declare that the research was conducted in the absence of any commercial or financial relationships that could be construed as a potential conflict of interest.

## Publisher’s Note

All claims expressed in this article are solely those of the authors and do not necessarily represent those of their affiliated organizations, or those of the publisher, the editors and the reviewers. Any product that may be evaluated in this article, or claim that may be made by its manufacturer, is not guaranteed or endorsed by the publisher.
